# Exploring oxygen-affinity-controlled TaN electrodes for thermally advanced TaO_x_ bipolar resistive switching

**DOI:** 10.1038/s41598-018-26997-y

**Published:** 2018-06-04

**Authors:** Taeyoon Kim, Gwangho Baek, Seungmo Yang, Jung Yup Yang, Kap Soo Yoon, Soo Gil Kim, Jae Yeon Lee, Hyun Sik Im, Jin Pyo Hong

**Affiliations:** 1 Novel Functional Materials and Devices Lab, The Research Institute of Natural Science, Department of Physics, Hanyang University, Seoul, 04763 South Korea; 2 Division of Nanoscale Semiconductor Engineering, Hanyang University, Seoul, 04763 South Korea; 3 Department of Physics, Kunsan National University, Geonbuk, 54150 South Korea; 4 SK Hynix Semiconductor Inc., Icheon, 17336 South Korea; 5 Department of Semiconductor Science, Dongguk University, Seoul, 04620 South Korea

## Abstract

Recent advances in oxide-based resistive switching devices have made these devices very promising candidates for future nonvolatile memory applications. However, several key issues remain that affect resistive switching. One is the need for generic alternative electrodes with thermally robust resistive switching characteristics in as-grown and high-temperature annealed states. Here, we studied the electrical characteristics of Ta_2_O_5−x_ oxide-based bipolar resistive frames for various TaN_x_ bottoms. Control of the nitrogen content of the TaN_x_ electrode is a key factor that governs variations in its oxygen affinity and structural phase. We analyzed the composition and chemical bonding states of as-grown and annealed Ta_2_O_5−x_ and TaN_x_ layers and characterized the TaN_x_ electrode-dependent switching behavior in terms of the electrode’s oxygen affinity. Our experimental findings can aid the development of advanced resistive switching devices with thermal stability up to 400 °C.

## Introduction

A long-standing goal in the development of potential next-generation nonvolatile memory (NVM) devices is to meet the demands of key memory markets, which include high scalability for high density integration and fast operation speed. Among recently considered NVMs, those with resistive switching have shown promise for use in nanoscalable resistive random access memory (ReRAM)^1^ with an ideal 4F^2^ memory size. Numerous materials have been considered as storage media in ReRAM devices, including solid electrolytes, binary oxides, organic materials, and perovskites^2–5^. Various binary metal oxides such as AlO_x_^6^, TaO_x_^7–10^, ZrO_x_^11^, TiO_x_^12^, and HfO_x_^13^ have been identified as main data storage media due to their excellent endurance of more than 10^12^ cycles^14^. Although numerous studies have investigated the possibility of using these materials (with manipulation of material thickness^15^, electrodes^16,17^, heat treatment^18^, multi-layer frames^19^, and doping^20^), oxide reactions at the metal electrode/oxide interfaces in as-grown and annealed states still critically affect the characteristics of resistive switching^21^. This has restricted the choice of electrode because of the requirement for high temperature (~400 °C) at the back end of the line.

Interfacial reactions are widely believed to profoundly affect the characteristics of resistive switching^22^. Previous studies reported metal electrode-dependent resistive switching^16,22,23^, where electrodes with different work functions and oxygen affinities yielded different resistive switching characteristics. For example, even for resistive switching devices with the same metal oxide layer, the switching type and behavior were found to vary depending on electrode type^23^. Although diverse resistive switching features are linked to the oxygen affinities of electrodes governing the interfacial reaction between the electrode and oxide layer^17^, the effect of varying the oxygen affinities of the electrode materials has not yet been comprehensively examined.

In this work, we address the resistive switching characteristics of as-grown and annealed Ta_2_O_5−x_-based ReRAM devices with bottom TaN_x_ electrodes containing different amounts of nitrogen. Precise control of nitrogen content in TaN_x_ during growth was used to achieve adjustable structural phases in the TaN_x_ layer, ranging from metallic body-centered cubic (bcc) TaN_x_ or faced-centered cubic (fcc) TaN_x_ to a mixture of fcc TaN_x_ with N-rich phases. Capacitance-voltage (C-V) and X-ray photoelectron spectroscopy (XPS) measurements at the interfacial layer demonstrated diverse oxygen affinities, which resulted in discrete switching events. Experimental findings suggest that low oxygen-affinity TaN_x_ electrodes are essential for ensuring thermally stable resistive switching responses in annealed states.

## Results and Discussion

### Structural and electrical characteristics of the TaN_x_ layer

**Figure 1:**
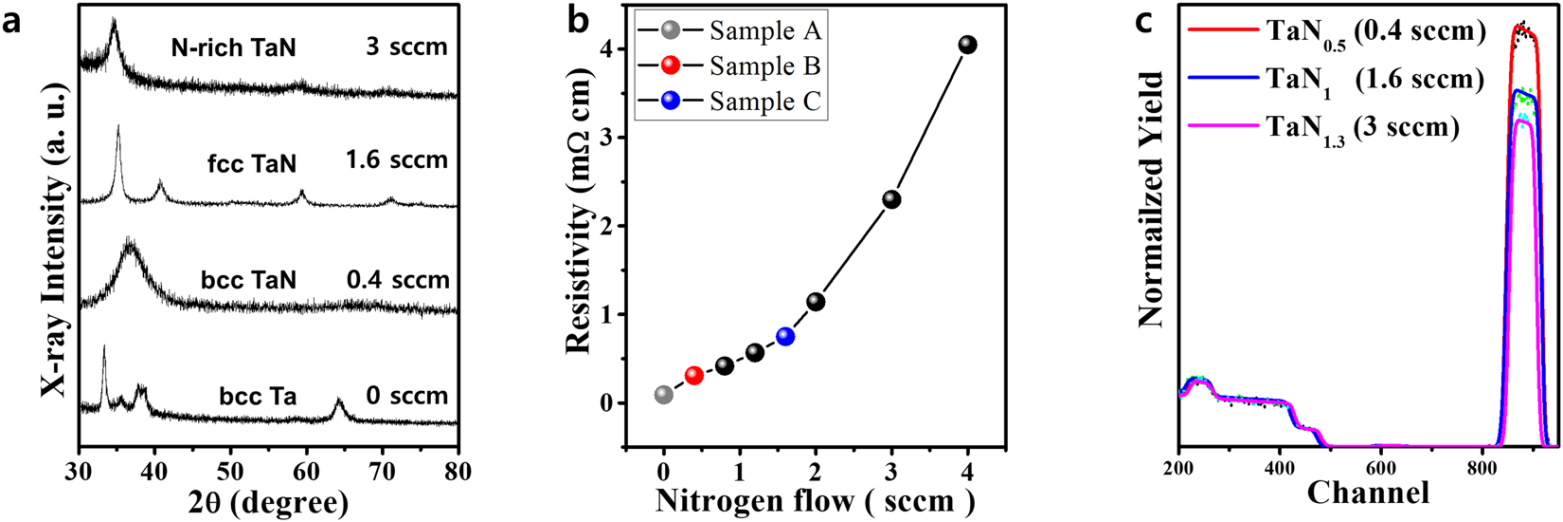
Nitrogen flow rate contribution to the structural/electrical characteristics of TaN_x_ layers. (**a**) Representative XRD θ-2θ plots of TaN_x_ layers for various nitrogen flow rates during growth. Corresponding variation in (**b**) resistivity and (**c**) Ta/N composition determined by RBS analyses, where each colored line in Fig. 1c represents Ta/N compositions in the grown TaN_x_ layers, for three nitrogen flow rates: 0.4 sccm (red), 1.6 sccm (blue), and 3 sccm (purple). Note that resistivity and N composition increased with increasing nitrogen flow rate during growth.

Representative X-ray diffraction (XRD) patterns for Ta and as-grown TaN_x_ layers for nitrogen flows in the 0–3 sccm range are shown in Fig. 1a. The pure Ta layer exhibited a typical sharp (200) peak at ~33°, confirming the presence of a bcc crystal structure (PDF card No: 01-089-4763). The flow rate of 0.4 sccm resulted in a broad peak that may be associated with closely-packed (110) faces at around 37°, possibly indicating the formation of bcc TaN_x_^24^ (PDF card No: 00-025-1278). However, another possibility is that mixed phase (110) bcc Ta(N) and (101) hexagonal Ta_2_N may have been present. Further increasing the nitrogen flow to the 1.2–2 sccm range yielded four clear peaks [(111), (200), (220), and (311)] in the TaN_x_ layer, implying the presence of fcc TaN_x_ (PDF card No: 04-014-0112). The flow rate of 3 sccm appeared to yield a nitrogen-rich fcc TaN_x_ structure (PDF card No: 01-079-5806). Thus, suitable control of the nitrogen content during growth induced the formation of various TaN_x_ crystal structures^25^. These XRD findings were consistent for all samples in our experiments. Figure 1b shows the resistance curves for the as-deposited TaN_x_ layers that were measured using a standard four-point method. Resistance tended to increase with increasing nitrogen flow rate during growth and increased sharply for flow rates above 3 sccm. The resistivities of Ta, TaN_x_ (0.4 sccm), TaN_x_ (1.6 sccm), and TaN_x_ (3 sccm) layers were determined to be 0.18 mΩcm, 0.26 mΩcm, 0.34 mΩcm, and 1.13 mΩcm, respectively.

Previous studies have shown that, as the nitrogen content inside the TaN layer increases, crystallinity in the (111) peak of TaN layer increases^26^ or the lattice constant increases^27^, resulting in increased resistance of the TaN layer.

Thus, in our experiments, we chose three different TaN_x_ bottom electrodes to determine the electrode-type dependence of resistive switching characteristics; for these chosen electrodes, the nitrogen flows during growth were 0.4, 1.6, and 3 sccm. The 3-sccm-flow TaN_x_ electrode was excluded from these experiments because of its higher resistance. Figure 1c shows the Rutherford back scattering spectroscopy (RBS) analyses of TaN_x_ (0.4 sccm), TaN_x_ (1.6 sccm), and TaN_x_ (3 sccm) layers; nitrogen-to-tantalum stoichiometric ratios of 0.5, 1, and 1.3, respectively, were identified.

**Figure 2:**
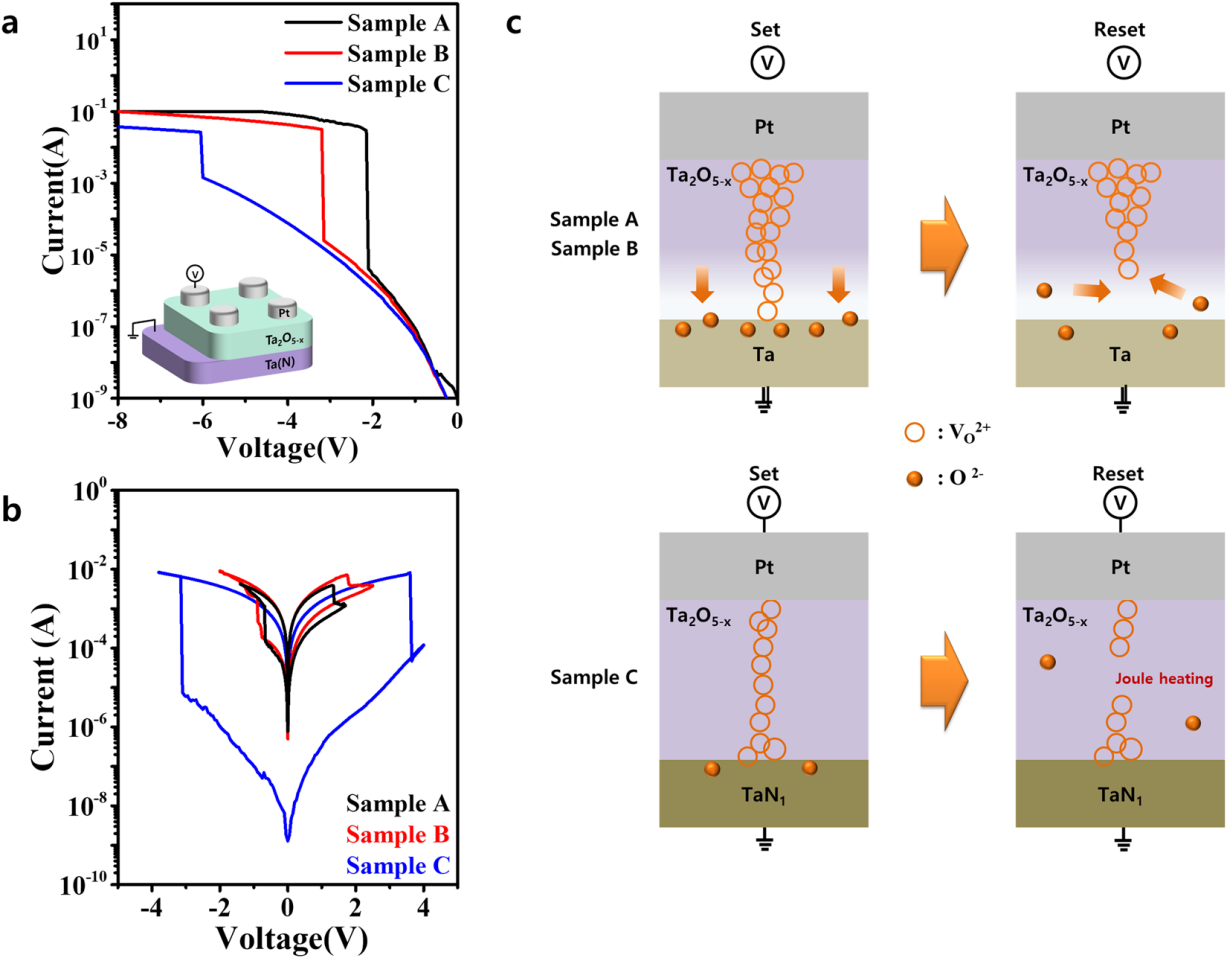
(**a**) Electrical forming I-V characteristics of Samples A, B, and C, which were designed to have a Ta, TaN_0.5_, and TaN_1_ layer as a bottom electrode, respectively. The inset in Fig. 2a schematically shows the Pt/Ta_2_O_5−x_ /TaN_x_ cell. Increasing the nitrogen content of the TaN_x_ electrode increased the forming voltage. (**b**) The three I-V curves show the bipolar resistive switching responses of Samples A, B, and C. Sample C clearly exhibited a larger on/off ratio (>10^3^) and larger set/reset voltage than Samples A and B. (**c**) Possible switching behaviors of Samples A/B and C; arrows and circles represent bias-dependent oxygen ion drift and corresponding conducting filaments and oxygen vacancies, respectively.

Three samples were prepared: Pt/Ta_2_O_5−x_ (20)/Ta (Sample A), Pt/Ta_2_O_5−x_ (20)/TaN_0.5_ (Sample B), and Pt/Ta_2_O_5−x_ (20)/TaN_1_ (Sample C), where the numbers in parentheses are the nominal thicknesses of the corresponding active layers in nanometers. Samples A, B, and C were designed to have Ta (bcc), TaN_0.5_ (bcc), and TaN_1_ (fcc) layers, respectively, as bottom electrodes. Figure 2a shows the electrical forming I-V characteristics of Samples A, B, and C, and typical schematics of resistive switching devices are shown in the inset of Fig. 2a. All samples required a negative forming voltage for clear bipolar resistive switching. In addition, as seen in this figure, a relatively high forming voltage was needed for the TaN_x_ electrodes containing nitrogen, because of their inherent resistivity induced by the nitrogen content in the Ta layer compared with the pure Ta electrode. Figure 2b shows representative I-V curves for Samples A, B, and C for 100 consecutive cycles. All samples exhibited typical bipolar resistive switching. That is, after forming, their resistive states switched to low resistance states (LRSs). When a voltage sweep from zero in the negative direction was applied to the top electrode, a sudden reduction in resistance, from the high resistance state (HRS) to the LRS, was observed. As the applied voltage was swept from negative values to zero, the LRS was maintained in all of the samples. When the applied voltage was swept from zero to positive values, an increase in resistance from the LRS to the HRS was observed. As the applied voltage was swept from positive values to zero, the HRS was maintained. During this resistive switching, Samples A and B exhibited similar resistive switching characteristics, including similar set/reset voltages and on/off current ratios (~10^1^). However, Sample C clearly exhibited a larger set/reset voltage and a higher on/off current ratio (>10^3^) than Samples A and B. This indicates that the characteristics of the bottom electrode of Sample C were different from those of Samples A and B. However, even though Samples A/B and C exhibited different behaviors, the observed bipolar resistive switching behavior can be understood in terms of the translocation of oxygen ions or vacancies^28^.

The two different resistive switching characteristics of Samples A and C can be explained by the VCM model^29^ (Sample B is excluded because of its similar switching behaviors to Sample A); these mechanisms are illustrated in Fig. 2c. First, in Sample A, a negative forming voltage generated mobile oxygen ions in the Ta_2_O_5−x_ layer. In the presence of an electrical field, these mobile oxygen ions drifted toward the Ta bottom electrode and localized near the Ta_2_O_5−x_/Ta interface because of the higher oxygen affinity of the Ta electrode. Simultaneously, oxygen vacancies migrated to the top Pt electrode, enabling the formation of a conical filament within the Ta_2_O_5−x_ film that grew toward the Ta bottom electrode. The cell switched to LRS when the conducting filament extended completely through the metal oxide material. Applying a positive bias to the top electrode released the oxygen ions accumulated in the interfacial region, causing a gradual reset process associated with bias-dependent gradual annihilation of the filament. Sample C exhibited a higher forming voltage than Sample A. This may be due to the existence of a small number of oxygen vacancies inside the Ta_2_O_5−x_ layer because of the relatively lower oxygen affinity of the TaN_x_ electrode used in Sample C; oxygen affinity will be discussed later. Thus, a positive bias to the top electrode of Sample C easily ruptured the weakest pre-existing filamentary paths provided by a high-voltage-electroforming process through Joule heating-assisted electrochemical dissolution of the filaments rather than electro-chemical migration of oxygen^30^. Joule heating-assisted electrochemical dissolution process in the weakest parts of the pre-existing filamentary paths of Sample C appeared to cause the sharp reset event with a 10^3^–10^4^ on/off ratio. An additional performance analysis of Samples A and C is provided in Figs S1 and S2.

Another study suggested that resistive switching in ReRAM devices was closely related to the oxygen affinity and metal work function of the electrodes^17^. To further investigate the difference between the oxygen affinities of various TaN_x_ electrodes, capacitance-voltage analysis was performed for Samples A, B, C, and D (Pt/TaO_x_/Pt). After the C-V measurements, voltage linearity was estimated from the value of the quadratic parameter α, as follows:

1 $$\frac{{\rm{\Delta }}C}{{C}_{0}}=\frac{C(V)-{C}_{0}}{{C}_{0}}=\alpha {V}^{2}+\beta V$$

**Figure 3:**
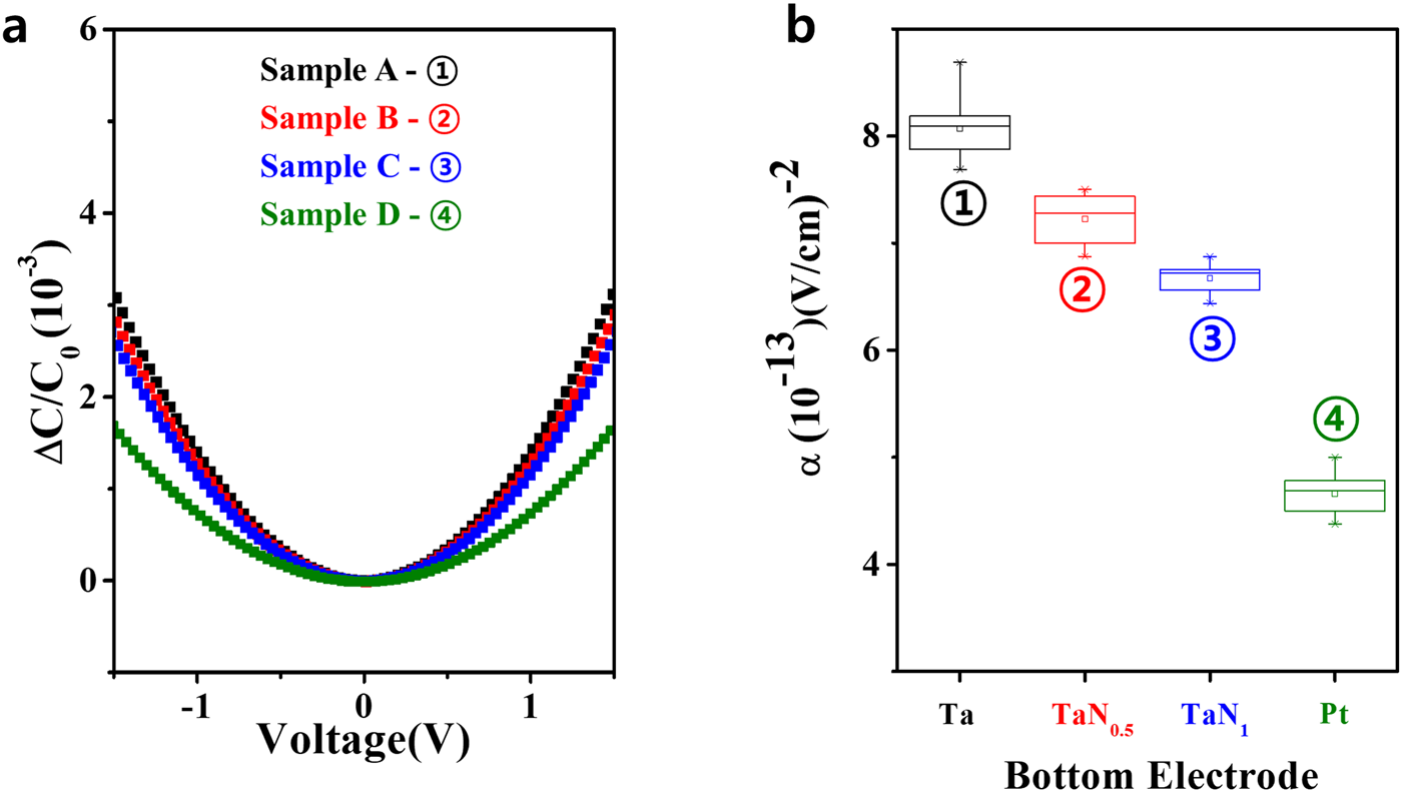
Electrode-dependent capacitances and corresponding oxygen affinities of TaN_x_ layers. (**a**) Variation in capacitance (ΔC/C_0_) acquired at 100 kHz as a function of dc voltage. (**b**) Quadratic voltage coefficient of capacitance as a function of the bottom electrode type; results were subsequently fitted using the equation provided in the main text. The observed variation in α reflects the formation of electrode-dependent thin interfacial layers and the corresponding oxygen affinity of each TaN_x_ bottom electrode. A low oxygen affinity is indicative of a low redox reaction with an oxide active layer used in a memory cell. Note that Pt and Ta electrodes clearly and expectedly demonstrate low and high values of α, a finding that can be ascribed to their low and high oxygen affinities, respectively.

Here, C_0_ is the capacitance at zero bias, V is the DC applied bias, and α and β are the quadratic and linear voltage coefficients of capacitance, respectively. The linear term β captures the asymmetry of carrier depletion or injection in the dielectric in the top and bottom electrodes. Variations in α have been correlated with the formation of electrode-dependent thin interfacial layers^31–33^. In general, it is well-known that oxygen vacancies affect resistive switching^34^, and the oxygen affinity values of various electrodes are strongly associated with α value. A previous study also showed that the magnitude of α corresponds to the electrode oxygen affinity, which was confirmed based on the heat of formation energy for each electrode^35^. Thus, we determined the difference between the oxygen affinities of various TaN_x_ electrodes by measuring the α using C-V. Figure 3a shows the normalized capacitance ΔC/C_0_ as a function of the DC electric field applied to Samples A, B, C, and D. These curves yield α from second order polynomial fits, as shown in Fig. 3b. The α is given as a function of the bottom electrode, and variation in α reflects both the formation of electrode-dependent thin interfacial layers and the oxygen affinity of each TaN_x_ bottom electrode^33^. A low oxygen affinity is indicative of a low redox reaction, which is frequently observed in oxide active layer-based ReRAM devices. As expected, the Pt and Ta electrodes in particular demonstrated low and high α values, indicating low and high oxygen affinities, respectively. These C-V findings indicate that increasing the nitrogen content of the TaN_x_ electrode decreases α, corresponding to a lower oxygen affinity.

**Figure 4:**
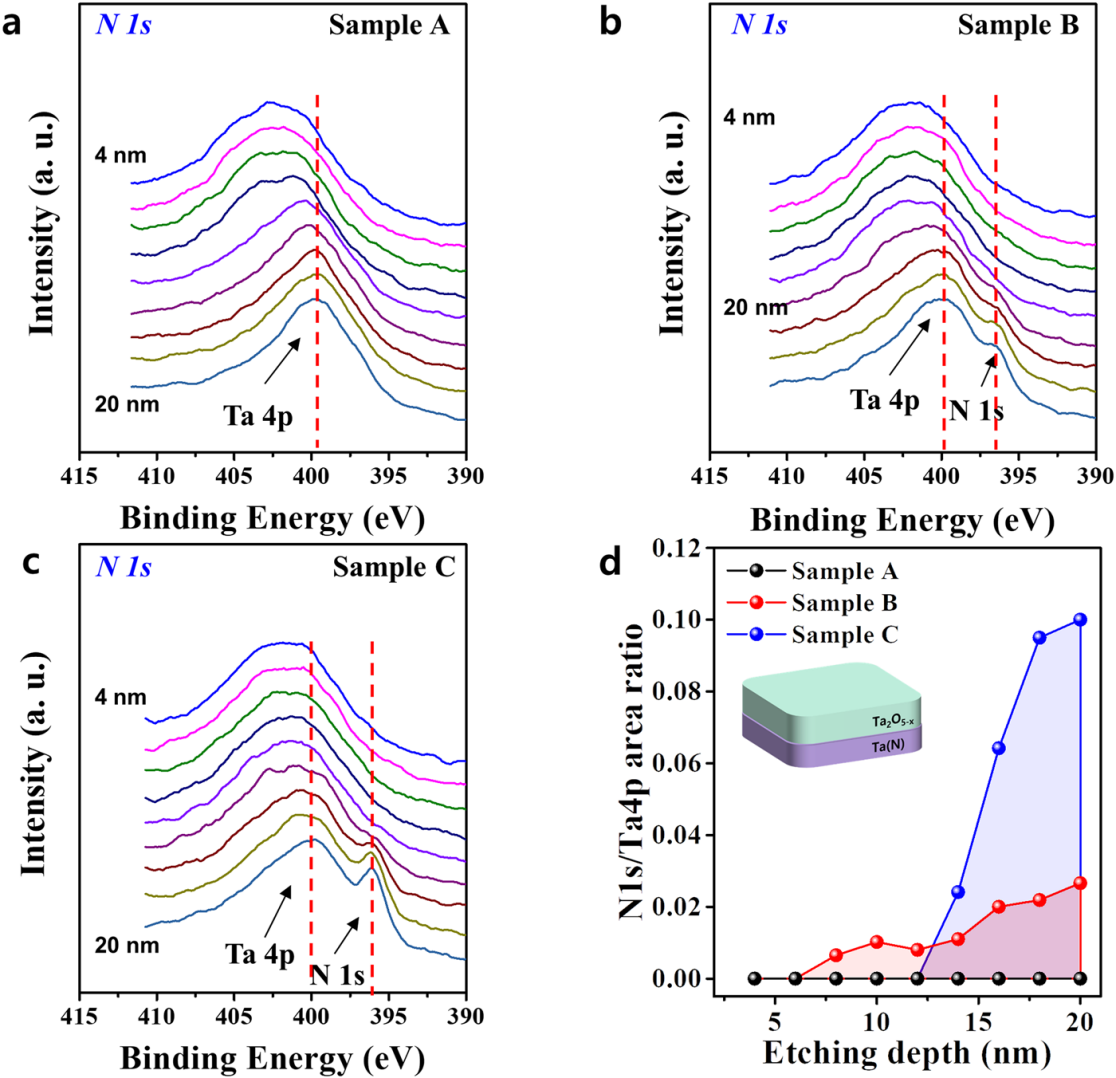
XPS binding energy depth profiles and summarized nitrogen diffusivity for N 1 s peaks. (**a**) Sample A, (**b**) Sample B, and (**c**) Sample C, ranging from 4 nm to 20 nm into the Ta_2_O_5−x_ layer. The N 1 s spectra were de-convoluted into two different peaks: Ta 4p and N 1 s. (**d**) Summary of the variation in nitrogen contents with depth in the Ta_2_O_5−x_ oxide layer for N1s/Ta4p peak areas ratio (**a**–**c**); the inset shows a schematic of the XPS measurements. The TaN_x_ electrode used in Sample C had a larger area ratio of N1s/Ta4p than that in Sample B. However, the diffusivity of nitrogen atoms was greater for Sample B than Sample C, establishing unstable resistive switching compared with Sample C.

To further clarify the composition and chemical bonding states of Samples A, B, and C, chemical bonding states associated with oxygen deficiencies were examined using XPS depth profile analysis. XPS spectra were calibrated using the binding energy of the C 1 s peak (285.5 eV) as a reference, and the Ar sputtering etching rate was ~0.2 nm/s and ranged from 4 nm to 20 nm in the Ta_2_O_5−x_ layer. Figure 4a–c show representative XPS binding energy depth profiles for the N 1 s core level, revealing a clear transitional region at the Ta_2_O_5−x_/ TaN_x_ interface. In this figure, the N 1 s binding energy for each case was de-convoluted into two peaks, corresponding to a high binding peak (Ta 4p) and a low binding peak (N 1 s)^36,37^. Diffusion of nitrogen atoms from the TaN_x_ electrode into the Ta_2_O_5−x_ layer was observed. Nitrogen contents represented by N1s/Ta 4p peak area ratio were plotted as a function of depth in the Ta_2_O_5−x_ oxide layer (Fig. 4d) along with a conceptual sketch of the XPS measurements. As shown in Fig. 4d, the content of N 1s for Sample C was larger than that for Sample B, but the diffusivity of nitrogen atoms in Sample C was lower than that for Sample B. We assumed that the lower diffusion of N atoms from the TaN electrode toward the Ta_2_O_5_ layer in Sample C than Sample B was due to stronger binding between the Ta and N elements in Sample C. Previous work showed that the fcc TaN_x_ layer frequently serves as a diffusion barrier^38^ because of strong binding between tantalum and nitrogen, thus improving the thermal stability of Sample C. As described above, the resistive switching behaviors of Samples A and B likely arose from typical electrochemical redox reactions induced by the higher oxygen affinities observed in the bottom Ta and TaN_0.5_ electrode (observed for conventional ReRAM devices). That is, under a negative applied voltage, oxygen ions migrated to the bottom TaN_x_ electrode and were stored in the interfacial layer. In this situation, the bottom electrode serves as a reservoir for mobile oxygen ions. When oxygen ions were extracted near the interface of the Ta_2_O_5−x_ layer, gradient oxygen vacancies formed in the Ta_2_O_5−x_ layer and generated a conductive filament^39^. However, the low diffusivity of Sample C containing the bottom fcc TaN_x_ electrode limited the number of oxygen ions that migrated into the bottom electrode from the Ta_2_O_5−x_ layer. That is, because the ability of the TaN_1_ electrode of Sample C to absorb oxygen atoms was far lower than that of the Ta electrode of Sample A, oxygen ions did not migrate into the bottom electrode from the Ta_2_O_5−x_ layer. These results indicate the presence of an almost stoichiometric Ta_2_O_5−x_ layer in Sample C, reflecting a relatively high forming voltage for the formation of a conductive filament in the Ta_2_O_5−x_ layer, as seen in Fig. 2a. High forming voltage-driven filaments often reflect very low resistivity, which is also known as the soft-breakdown process^40^. In this process, a very strong current flowing through filaments with relatively low resistivity causes severe Joule heating, manifesting as a sharp reset event during the reset process^41^. This may explain why resistive switching devices with low oxygen affinity bottom electrodes exhibit sharp set/reset resistive switching.

### Contribution of annealing to resistive switching responses

**Figure 5:**
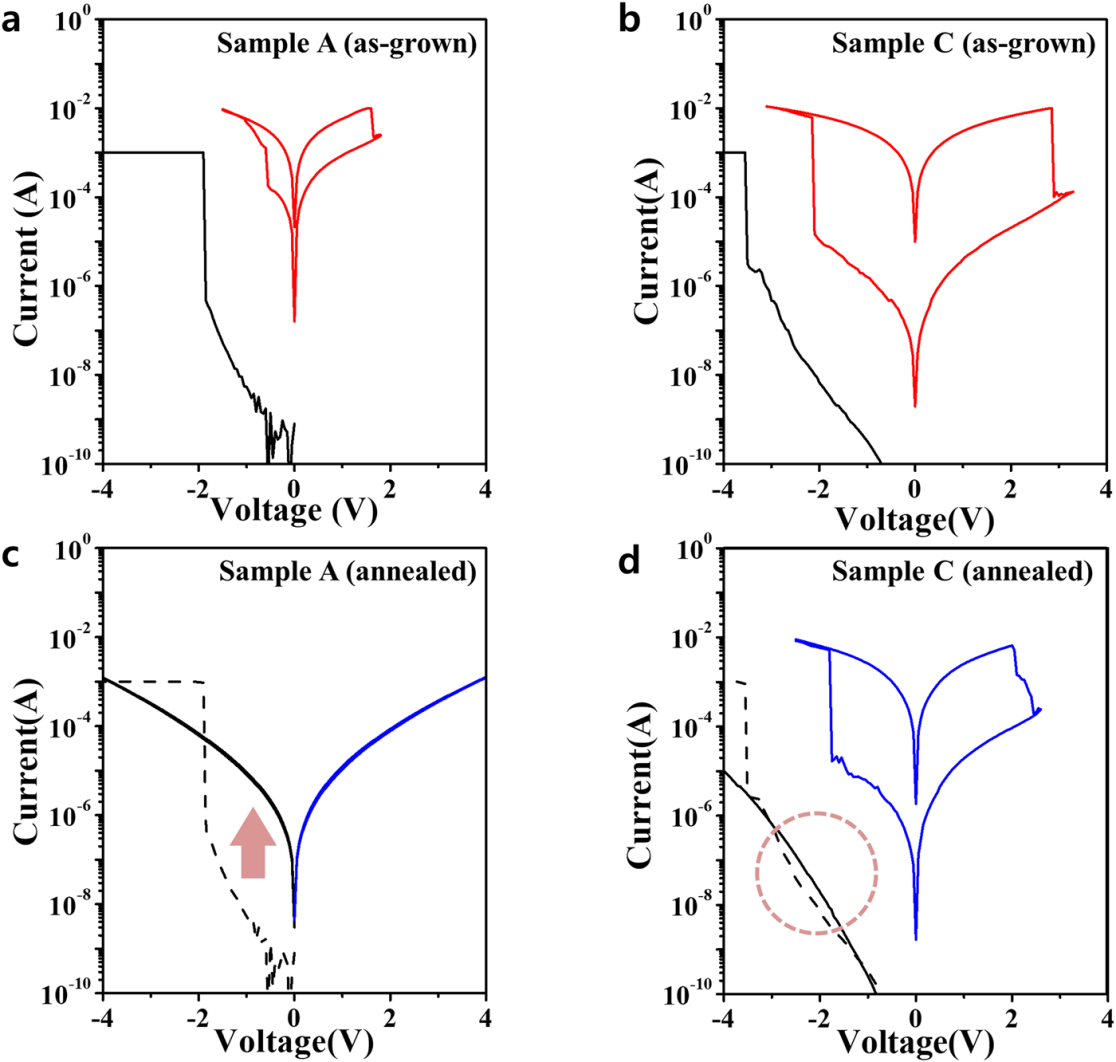
Resistive switching characteristics of I-V curves for (**a**) Sample A (as-grown), (**b**) Sample C (as-grown), (**c**) Sample A (annealed), and (**d**) Sample C (annealed). Annealed Sample A exhibited a decrease in initial resistance and loss of resistive switching behavior because of the presence of a large amount of oxygen vacancies after the annealing process. However, initial resistance and resistive switching behaviors of Sample C were almost unchanged, demonstrating the robust thermal stability, where the amount of oxygen vacancies in the Ta_2_O_5−x_ film remained almost unchanged, even after post-annealing at 400 °C.

Resistive switching measurements and XPS analyses were also conducted for Samples A and C to gain insight into the differences between the resistive switching characteristics of these samples after post-annealing at 400 °C for 1 h in air. Figure 5 shows bipolar resistive switching responses for (a) Sample A (as-grown), (b) Sample C (as-grown), (c) Sample A (annealed), and (d) Sample C (annealed). The annealed Sample A with an interfacial TaO_x_ layer exhibited a decrease in initial resistance, and resistive switching did not occur after 400 °C post-annealing; we attributed this to an increase in the number of oxygen vacancies induced by the reaction of Ta layer with the oxygen in the Ta_2_O_5_ layer. In contrast, Sample C with a relatively low oxygen affinity had little reaction with the Ta_2_O_5_ layer before and after the thermal annealing process. Thus, the resistive switching for Sample C remained almost unchanged even after post-annealing at 400 °C, providing evidence of thermal stability up to 400 °C (Supplementary Figure S2). Similar results were observed upon thermal annealing in a nitrogen atmosphere. Detailed I-V characteristics of Samples A and C are provided in Figure S3 (Supplementary Information). Together, these post-annealing observations imply that the observed differences between the interfacial layers of Samples A and C are associated with oxygen vacancies generated by the annealing process.

**Figure 6:**
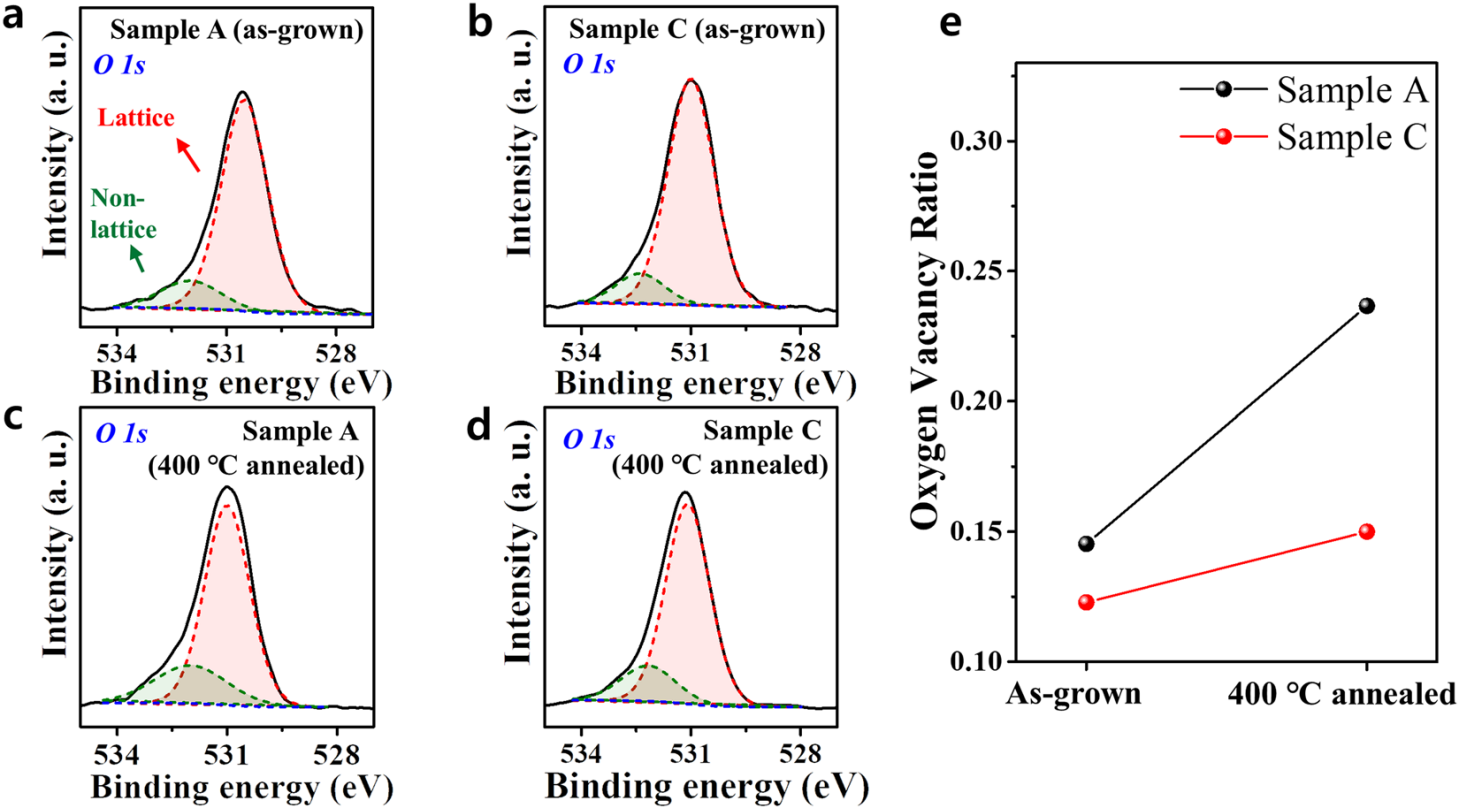
Oxygen vacancy ratio for as-grown/annealed Samples A and C. The O 1s XPS binding energy spectra of the Ta_2_O_5−x_ layer recorded for (**a**) Sample A (as-grown), (**b**) Sample C (as-grown), (**c**) Sample A (annealed at 400 °C) and (**d**) Sample C (annealed at 400 °C). The O 1s spectra were de-convoluted into two different peaks: red and green peaks. (**e**) Summarized oxygen vacancy ratio for Samples A and C. The observed oxygen vacancy ratio for Sample C remained almost unchanged, while that for Sample A revealed the presence of a relatively large ratio after annealing.

**Figure 7:**
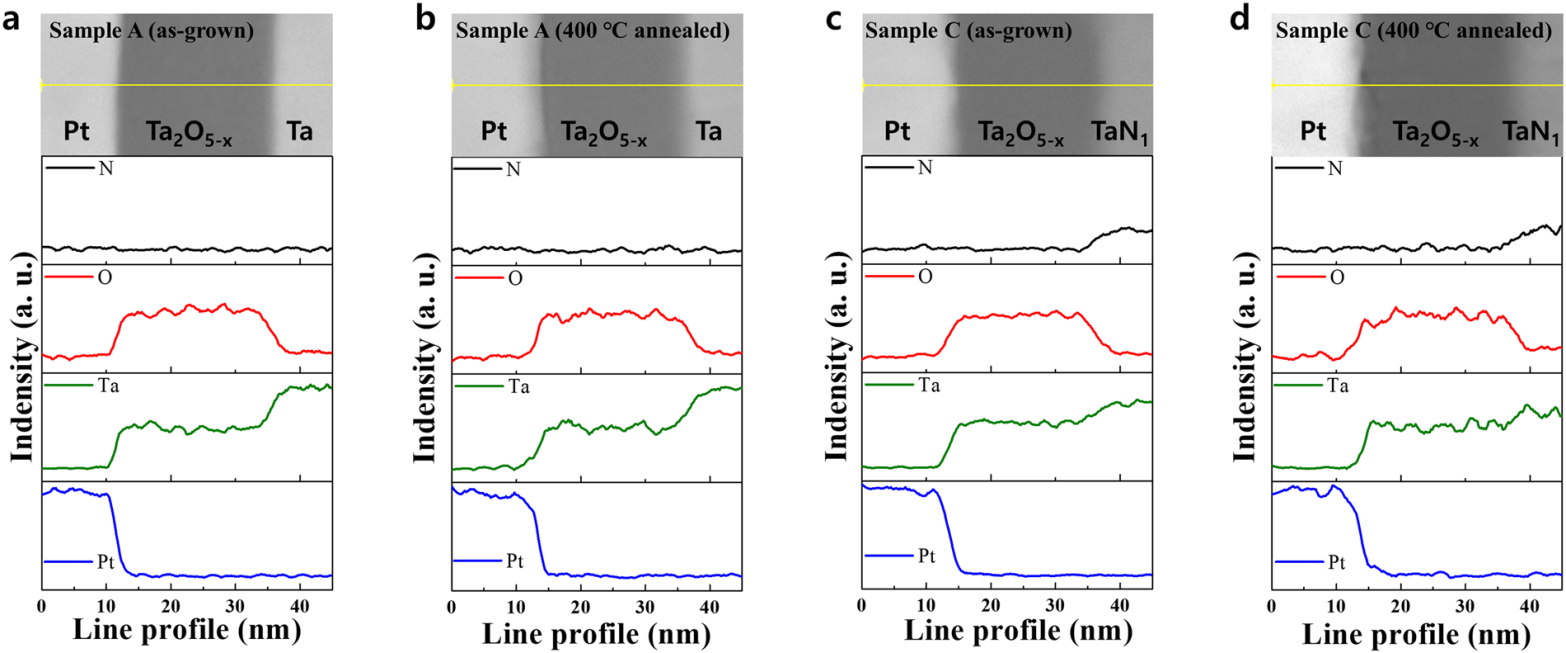
EDS line profiles and cross-sectional STEM images. (**a**) Sample A (as-grown), (**b**) Sample A (annealed at 400 °C), (**c**) Sample C (as-grown), and (**d**) Sample C (annealed at 400 °C). As seen in this figure, the interface of Sample C remains mostly unchanged, reflecting weak inter-diffusion of oxygen, while Sample A exhibited obvious enlargement of the oxygen diffusion layer at the interface, corresponding to strong inter-diffusion of oxygen during growth and annealing.

To elucidate the mechanisms underlying the annealing effect, a more thorough microstructural analysis of Ta_2_O_5−x_/interfacial layers of Samples A and C was performed by analyzing high-resolution (HR) transmission electron microscopy (TEM) data, energy dispersive spectroscopy (EDS) line profiles, and XPS depth profiles. Figure 6 shows XPS chemical state variation in the analyzed Ta_2_O_5−x_ layers, where the O 1 s XPS binding energy spectra of the Ta_2_O_5−x_ layers were recorded for (a) Sample A (as-grown), (b) Sample C (as-grown), (c) Sample A (annealed), and (d) Sample C (annealed). XPS measurement was carried out with a 20-nm-thick Ta_2_O_5_ sample, where each device also had the same 20 nm thickness. About 10 nm (half of the entire thickness) was etched by Ar plasma sputtering before XPS measurements. The O 1 s spectra of the Ta_2_O_5−x_ layers were de-convoluted to obtain two peaks at 530.5 eV at 532.0 eV^42–44^. The first oxygen peak depicts oxygen bonded in stoichiometric Ta_2_O_5_^43^, while the second oxygen peak indicates oxygen-deficient Ta_2_O_5−x_, reflecting the presence of unbonded oxygen molecules (oxygen interstitials) or oxygen vacancies. Thus, the concentrations of oxygen vacancies in the Ta_2_O_5−x_ films with/without the annealing process were estimated. As shown in Fig. 6e, the oxygen vacancy ratio for Sample C remained almost unchanged, while an increase in the amount of oxygen vacancies after annealing was observed for Sample A. This vacancy increase corresponded to a decrease in the initial resistance of the Ta_2_O_5−x_ layer, resulting in the absence of bipolar resistive switching after annealing, in good agreement with the results obtained by analyzing the I-V curves in Fig. 5. The similar resistive switching responses of Sample C before and after annealing imply that the fcc nature of TaN_x_ in Sample C is not significantly affected by annealing. Figure 7 shows the corresponding EDS analyses for Samples A and C before and after 400 °C annealing process, along with typical cross-sectional TEM images that exhibit multiple stacked configurations with clear interfaces. These experimental EDS results are consistent with TaN_x_ electrode-dependent diffusion of oxygen atoms during growth. As seen in Ta and O EDS profiles of Fig. 7 prior to and after annealing process, there seems to be an interfacial reaction at the Ta_2_O_5−x_/Ta interface at Sample A. However, the interface of Sample C remains mostly unchanged. Thus, the fcc TaN_x_ electrode used in Sample C exhibited sufficient thermal stability in our work.

## Conclusions

In summary, we investigated the resistive switching characteristics of as-grown and annealed Ta_2_O_5−x_-based devices with suitable TaN_x_ electrodes. Controlled variation of the nitrogen content of the TaN_x_ electrode induced variations in oxygen affinities and structural phases of the TaN_x_ electrode. A systematic analysis of composition and chemical bonding states for as-grown and annealed Ta_2_O_5−x_ and TaN_x_ layers, along with TEM images, indicated a reduction in the diffusion of oxygen ions, even after post-annealing. Experimental findings verified that the TaN_x_ (x = 1) sample exhibited better electrical and thermal stability with a high on/off current ratio than the other samples. Therefore, using an electrode with a suitable oxygen affinity may ensure thermally robust resistive switching in future three-dimensional stacked x-y crossbar frames.

## Methods

### Sample fabrication

Various Pt/Ta_2_O_5−x_/Ta, Pt/Ta_2_O_5−x_/TaN_0.5_, Pt/Ta_2_O_5−x_/TaN_1_, and Pt/Ta_2_O_5−x_/TaN_1.3_ resistive switching memory devices were fabricated on commercially available SiO_2_/Si substrates. The bottom TaN_x_ electrode was formed using a Ta metal target on SiO_2_ substrates via reactive radio-frequency sputtering under a processing pressure of 5 mTorr Ar/N_2_ at various N_2_ partial pressures. The Ar sputtering gas flow was maintained at 20 sccm, while N_2_ was varied from 0 sccm to 4 sccm for a series of Ta and TaN bottom electrodes. In our work, nitrogen flow rates were intentionally varied to produce TaN_x_ electrodes with different nitrogen contents. A 20-nm-thick Ta_2_O_5−x_ oxide layer was chosen as the main active medium for memory elements. Then, 50-nm-thick Pt top electrodes were formed by sputtering metal targets with a 50 μm × 50 μm cell size defined by photolithography and a lift-off process.

### Electrical characterization

DC electrical measurements were performed using a Keithley 4200 semiconductor parameter analyzer (Keithley 4200 SPA, Keithley Instruments, Inc.). The four-probe method was utilized to investigate the properties of TaN thin films. C-V measurements of oxygen affinity were performed using an Agilent 4294 A Precision Impedance Analyzer (Agilent Technologies, Inc.). A positive voltage bias was applied to the top electrode so that the bottom electrode (TaN_x_) was the cathode and the top electrode (Pt) was the anode. Measurements were performed in air.

## Electronic supplementary material

Supplementary Information

## References

[1] Waser R, Dittmann R, Staikov G, Szot K (2009). Redox-Based Resistive Switching Memories - Nanoionic Mechanisms, Prospects, and Challenges. Adv. Mater..

[2] Suri M (2013). Bio-Inspired Stochastic Computing Using Binary CBRAM Synapses. IEEE Trans. Electron Devices.

[3] Pyun M (2008). Electrical and reliability characteristics of copper-doped carbon (CuC) based resistive switching devices for nonvolatile memory applications. Appl. Phys. Lett..

[4] Deleruyelle D (2013). Ge2Sb2Te5 layer used as solid electrolyte in conductive-bridge memory devices fabricated on flexible substrate. Solid State Electronics.

[5] Liu Y (2016). Resistive switching memory based on organic/inorganic hybrid perovskite materials. Vacuum.

[6] Zhang Y (2013). Study of conduction and switching mechanisms in Al/AlOx/WOx/W resistive switching memory for multilevel applications. Appl. Phys. Lett..

[7] Ninomiya T (2013). Improvement of Data Retention During Long-Term Use by Suppressing Conductive Filament Expansion in TaOx Bipolar-ReRAM. IEEE Electron Device Lett..

[8] Chakrabarti S (2017). Scalable cross-point resistive switching memory and mechanism through an understanding of H2O2/glucose sensing using an IrOx/Al2O3/W structure. Physical Chemistry Chemical Physics.

[9] Chakrabarti S (2018). Evolution of resistive switching mechanism through H2O2 sensing by using TaOx-based material in W/Al2O3/TaOx/TiN structure. Applied Surface Science.

[10] Samanta S, Maikap S, Roy A, Jana S, Qiu J-T (2017). Effects of W/Ir Top Electrode on Resistive Switching and Dopamine Sensing by Using Optimized TaOx-Based Memory Platform. Adv. Mater. Interfaces.

[11] Lee, J. *et al*. Effect of ZrO_x_/HfO_x_ bilayer structure on switching uniformity and reliability in nonvolatile memory applications. *Appl*. *Phys*. *Lett*. **97**, (2010).

[12] Baik SJ, Lim KS (2010). Bipolar resistance switching driven by tunnel barrier modulation in TiOx/AlOx bilayered structure. Appl. Phys. Lett..

[13] Chen, Y.-S. *et al*. An Ultrathin Forming-Free HfO_x_ Resistance Memory With Excellent Electrical Performance. *IEEE Electron Device Lett*. **31**, 1473–1475.

[14] Torrezan AC, Strachan JP, Medeiros-Ribeiro G, Williams RS (2011). Sub-nanosecond switching of a tantalum oxide memristor. Nanotechnology.

[15] Ito D, Hamada Y, Otsuka S, Shimizu T, Shingubara S (2015). Oxide thickness dependence of resistive switching characteristics for Ni/HfOx/Pt resistive random access memory device. Jpn. J. Appl. Phys..

[16] Xue WH (2014). Intrinsic and interfacial effect of electrode metals on the resistive switching behaviors of zinc oxide films. Nanotechnology.

[17] Chen C (2013). Migration of interfacial oxygen ions modulated resistive switching in oxide-based memory devices. J. Appl. Phys..

[18] Chen P-S, Lee H-Y, Chen Y-S, Chen F, Tsai M-J (2010). Improved bipolar resistive switching of HfOx/TiN stack with a reactive metal layer and post metal annealing. Jpn. J. Appl. Phys..

[19] Bae YC (2011). Oxygen Ion Drift-Induced Complementary Resistive Switching in Homo TiO x/TiO y/TiO xand Hetero TiO x/TiON/TiO xTriple Multilayer Frameworks. Adv. Funct. Mater..

[20] Guo T, Tan T, Liu Z, Liu B (2016). Oxygen vacancy modulation and enhanced switching behavior in HfOx film induced by Al doping effect. JOURNAL OF ALLOYS AND COMPOUNDS.

[21] Brivio S, Frascaroli J, Spiga S (2015). Role of metal-oxide interfaces in the multiple resistance switching regimes of Pt/HfO2/TiN devices. Appl. Phys. Lett..

[22] Waser R, Aono M (2007). Nanoionics-based resistive switching memories. Nat Mater.

[23] Pan F, Gao S, Chen C, Song C, Zeng F (2014). Recent progress in resistive random access memories: Materials, switching mechanisms, and performance. Materials Science and Engineering: R: Reports.

[24] Ohmi T, Sugawa S, Kotani K, Hirayama M, Morimoto A (2001). New paradigm of silicon technology. Proc. IEEE.

[25] Zaman A, Meletis E (2017). Microstructure and Mechanical Properties of TaN Thin Films Prepared by Reactive Magnetron Sputtering. Coatings.

[26] Nie HB (2001). Structural and electrical properties of tantalum nitride thin films fabricated by using reactive radio-frequency magnetron sputtering. Appl. Phys. A.

[27] Lee S, Lee YJ, Yoo MJ, Sun HJ (2016). Electrical Properties of Tantalum-nitride Films Prepared by Using Reactive Sputter Deposition. New Physics Sae Mulli.

[28] Lee AR (2016). Multifunctional resistive switching behaviors employing various electroforming steps. J. Mater. Chem. C.

[29] Lim E, Ismail R (2015). Conduction Mechanism of Valence Change Resistive Switching Memory: A Survey. Electronics.

[30] Strachan JP (2011). Spectromicroscopy of tantalum oxide memristors. Appl. Phys. Lett..

[31] Chiang KC (2007). High-Temperature Leakage Improvement in Metal Insulator Metal Capacitors by Work Function Tuning. IEEE Electron Device Lett..

[32] Cheng CH, Hsu HH, Chen WB, Chin A, Yeh FS (2010). Characteristics of Cerium Oxide for Metal–Insulator–Metal Capacitors. Electrochem. Solid-State Lett..

[33] Wenger C (2009). Influence of the electrode material on HfO_2_ metal-insulator-metal capacitors. J. Vac. Sci. Technol. B.

[34] Lee CB (2008). Effects of metal electrodes on the resistive memory switching property of NiO thin films. Appl. Phys. Lett..

[35] Vallée C, Gonon P, Jorel C, El Kamel F (2010). Electrode oxygen-affinity influence on voltage nonlinearities in high-k metal-insulator-metal capacitors. Appl. Phys. Lett..

[36] Zhou Q, Zhai J (2013). Study of the bipolar resistive-switching behaviors in Pt/GdOx/TaNx structure for RRAM application. phys. stat. sol. (a).

[37] Lee KB, Lee KH (2009). Optical Properties and X-ray Photoelectron Spectroscopy Study of Reactive-sputtered Ta-N Thin Films. J. Korean Phys. Soc..

[38] Zhao Y, Lu G (2009). First-principles simulations of copper diffusion in tantalum and tantalum nitride. Phys. Rev. B.

[39] Miao F (2011). Anatomy of a Nanoscale Conduction Channel Reveals the Mechanism of a High-Performance Memristor. Adv. Mater..

[40] Yao J, Zhong L, Natelson D, Tour JM (2011). Silicon Oxide: A Non-innocent Surface for Molecular Electronics and Nanoelectronics Studies. J. Am. Chem. Soc..

[41] Meng LY (2013). Impact of Joule heating on the microstructure of nanoscale TiO_2_ resistive switching devices. J. Appl. Phys..

[42] Wang G (2015). Three-Dimensional Networked Nanoporous Ta_2_O_5_-x Memory System for Ultrahigh Density Storage. Nano Lett..

[43] Huang CH, Chou T-S, Huang JS, Lin S-M, Chueh YL (2017). Self-Selecting Resistive Switching Scheme Using TiO_2_ Nanorod Arrays. Sci. Rep..

[44] Park S-J (2013). *In situ* control of oxygen vacancies in TiO_2_ by atomic layer deposition for resistive switching devices. Nanotechnology.

