# Supplementary material for: Exploring oxygen-affinity-controlled TaN electrodes for thermally advanced TaOx bipolar resistive switching
Source: Sci Rep. 2018 Jun 4;8:8532. doi: 10.1038/s41598-018-26997-y (PMC5986858; doi:10.1038/s41598-018-26997-y)
Supplement: Supplementary file 1 — Supplementary Information [file 41598_2018_26997_MOESM1_ESM.docx]

SUPPLEMENTARY INFORMATION

Correspondence and requests for materials should be addressed to J.H ([jphong@hanyang.ac.kr](mailto:jphong@hanyang.ac.kr))

**Exploring oxygen-affinity-controlled TaN electrodes for thermally advanced TaO_x_ bipolar resistive switching**

Tae Yoon Kim,^1^ Gwang Ho Baek,^2^ Seung Mo Yang, ^1^ Jung Yup Yang^3^, Kap Soo Yoon^1^, Soo Gil Kim^4^, Jae Yeon Lee^4^, Hyun Sik Im,^5^ and Jin Pyo Hong^1,2,*^

**1. Cumulative probabilities of as-grown Samples A and C.**


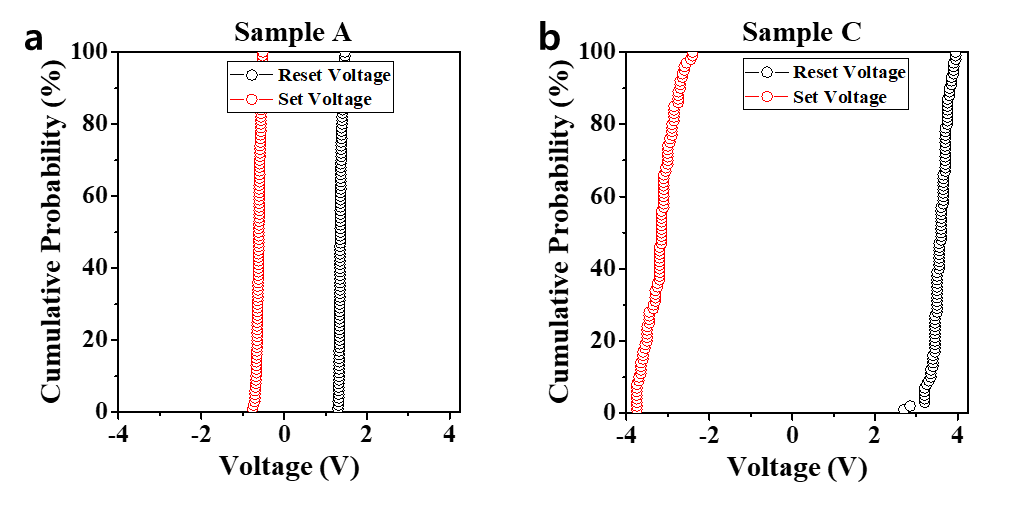


**Figure S1** Distributions of set/reset voltages for (a) Sample A and (b) Sample C during 100 consecutive switching cycles. Sample A had a narrow distribution of set/reset voltages, while Sample C had a relatively wide distribution of set/reset voltages, along with a stable resistive switching response.

**2. Comparison of endurance and retention features of Samples A and C.**

**
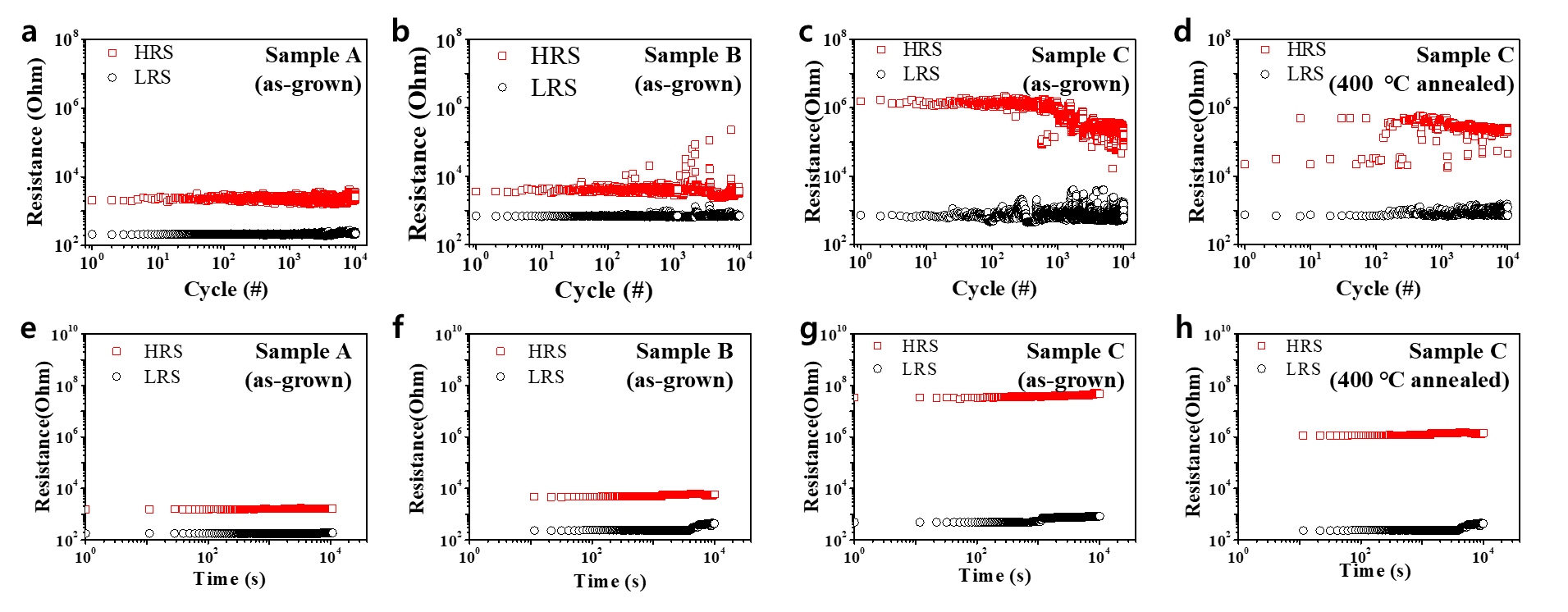
**

**Figure S2** Switching endurance analyses of (a) Sample A (as-grown), (b) Sample B (as-grown), (c) Sample C (as-grown), and (d) Sample C (annealed at 400°C) for 10^4^ operation cycles. Retention characteristics of (e) Sample A (as-grown), (f) Sample B (as-grown), (g) Sample C (as-grown), and (h) Sample C (annealed at 400°C) for 10^4^ s; the slight fluctuation in output for annealed Sample C indicates that more optimization work is required.

**3. Resistive switching behaviors of Samples A and C after the post-annealing process.**

**
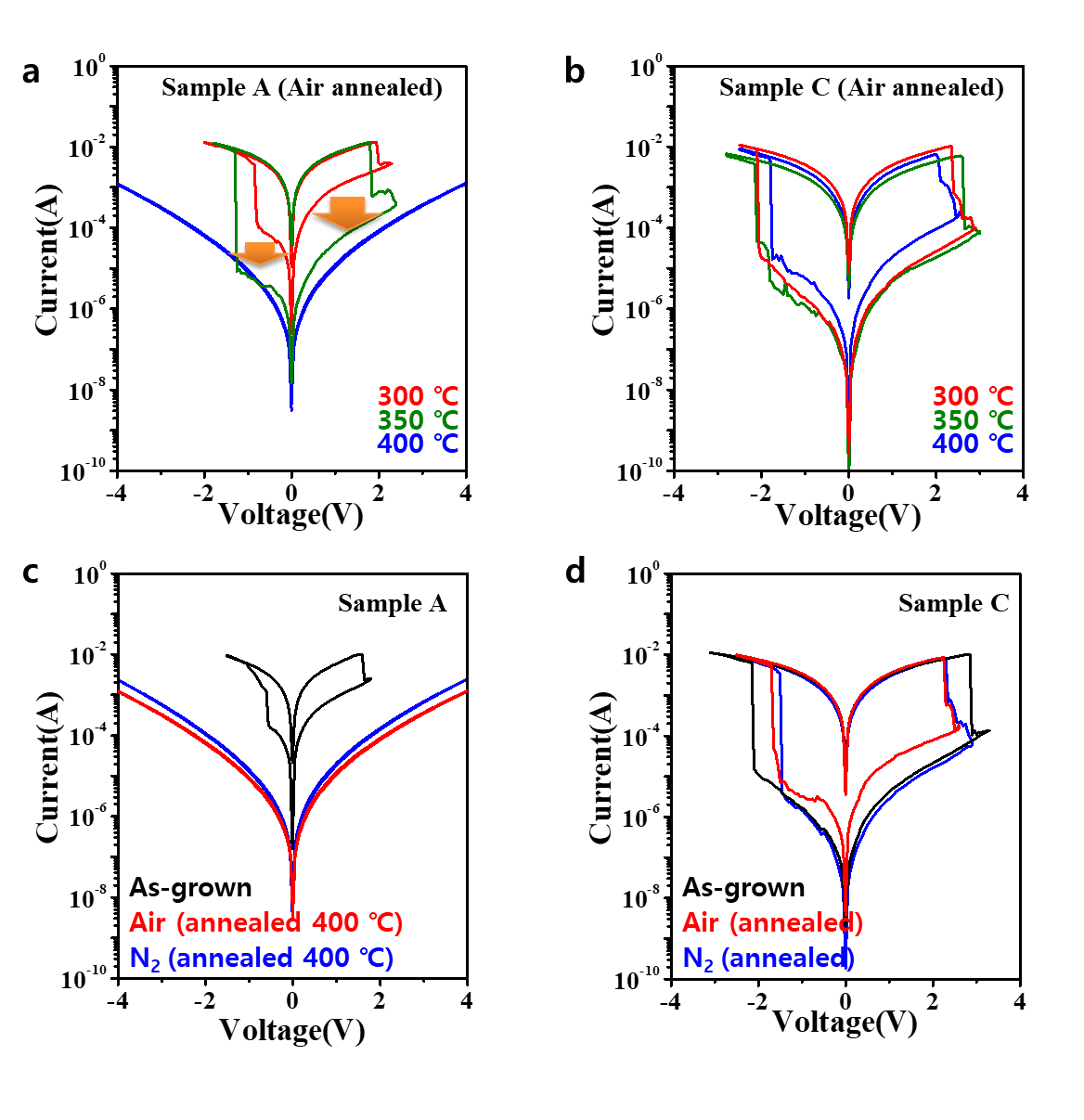
**

**Figure S3** Resistive switching characteristics of I-V curves for Samples A and C under two different conditions. First, Samples A (a) and C (b) were annealed in an air environment at various temperatures. Second, Samples A (c) and C (d) were annealed at 400 °C in air and N_2_ environments. Heat treatment was carried out at each temperature for 1 hour. Resistive switching behavior was absent in annealed Sample A. In contrast, the resistive switching behavior of Sample C was mostly unchanged, even after 400°C post-annealing, regardless of the annealing atmosphere.
